# Clinical response of pancreatic cancer bearing a germline BRCA2 p.I3169M fs^*^48 variant for platinum-based drug and PARP inhibitor

**DOI:** 10.1093/jjco/hyad157

**Published:** 2023-11-11

**Authors:** Risa Akahira, Koji Fukuda, Kazuhiro Shimazu, Taichi Yoshida, Daiki Taguchi, Hanae Shinozaki, Hiroshi Nanjyo, Hiroyuki Shibata

**Affiliations:** Department of Clinical Oncology, Graduaste School of Medicine, Akita University, Akita, Japan; Department of Clinical Oncology, Graduaste School of Medicine, Akita University, Akita, Japan; Department of Clinical Oncology, Graduaste School of Medicine, Akita University, Akita, Japan; Department of Clinical Oncology, Graduaste School of Medicine, Akita University, Akita, Japan; Department of Clinical Oncology, Graduaste School of Medicine, Akita University, Akita, Japan; Department of Clinical Oncology, Graduaste School of Medicine, Akita University, Akita, Japan; Department of Pathology, Akita University Hospital, Akita, Japan; Department of Clinical Oncology, Graduaste School of Medicine, Akita University, Akita, Japan

**Keywords:** pancreatic cancer, HBOC syndrome, PARP inhibitor, homologous recombination deficiency, BRCA2 p.I3169M fs^*^48

## Abstract

Pancreatic cancer is a malignancy with a high mortality rate, accounting for 37 000 people annually in Japan. It is rarely diagnosed in a resectable state, and effective medicines for its advanced stage are scarce. Some pancreatic cancer is hereditary, and ~10% have germline mutations of Breast cancer 1/2 (BRCA1/2). BRCA1/2 are key molecules involved in homologous recombination to repair DNA double-strand break. Platinum-based drugs and poly Adenosine diphosphate ribose (ADP) ribose polymerase inhibitors that induce synthetic lethality would be theoretically effective in patients with loss-of-function mutations in *BRCA1/2*. Strictly speaking, some discrepancy between the pathogenicity of *BRCA1/2* and their drug sensitivity might be expected. Hence, we report that platinum-based anticancer agents and poly ADP ribose polymerase inhibitors were effective against pancreatic cancer bearing BRCA2 p.I3169M fs^*^48.

## Teaser

BRCA2 is a key molecule involved in homologous recombination to repair DNA double-strand break. Pancreatic cancer bearing BRCA2 p.I3169M fs^*^48 is sensitive to platinum and poly ADP ribose polymerase inhibitors.

## Introduction

Pancreatic cancer is the seventh leading cause of cancer death globally. Japan reported 37 677 deaths in 2020, placing it in fourth place (1). The incidence rates widely vary among countries, with the highest age-adjusted incidence rates of 8.7–9.9 per 100 000 men and 5.8–7.2 per 100 000 women in Europe and North America and 7.0 per 100 000 men and 4.8 per 100 000 women in East Asia (2). In general, the incidence rate was higher in the developed countries than in the developing countries. Pancreatic neoplasms are histologically classified into two types, i.e. exocrine and endocrine types, depending on their origin; exocrine neoplasms derive from ductal or acinar cells and endocrine neoplasms from islet cells. Most (>95%) pancreatic cancers are exocrine, and ductal adenocarcinomas are the most abundant (80–85%) (3). In addition, familial pancreatic cancer accounts for at least 4–10% of pancreatic cancers (4). Moreover, only 10% of pancreatic adenocarcinomas are resectable at the time of diagnosis (4). Clinical Practice Guidelines recommend surgical treatment solely for patients with pancreatic cancer at clinical stages (cStage) 0, I and II, whereas cancers at late cStage II or cStage III are considered borderline resectable or unresectable due to the surrounding vascular invasion (5). Perioperative chemotherapy is advised for patients with cancers at cStage I to III, and continuous chemotherapy is offered individually for unresectable cStage IV cancer. However, there is a limited number of effective drugs available.

Gemcitabine plus nab-paclitaxel (GnP) is recommended as the first-line drug therapy for borderline resectable and unresectable pancreatic cancer (6). FOLFIRINOX, consisting of oxaliplatin (L-OHP), irinotecan (IRI) and continuous infusion of 5-fluorouracil (5FU), is an alternative first-line chemotherapy (6). The overall response rates were 37.9 and 69.9% in the GnP and FOLFIRINOX groups, respectively. The median progression-free survival (mPFS) was 6.7and 9.1 months in the GnP and FOLFIRINOX groups, respectively, for metastatic pancreatic cancer (6). The median overall survival (mOS) was 9.6 and 14.1 months in the GnP and FOLFIRINOX groups, respectively (6).

For these reasons, the 5-year survival rates remain low, ranging from 3 to 15%, even in developed countries (7). Therefore, people are waiting for the approval of new drugs against pancreatic cancer.

Until now, no molecular-targeted agents have been approved for pancreatic cancer treatment, except poly ADP ribose polymerase (PARP) inhibitors, which are effective for pancreatic cancer with *BRCA1/2* germline mutations (8). PARP acts in the base excision repair pathway responsible for single-strand breaks, which is the most common type of DNA damage (8). *BRCA1/2* genes are responsible for hereditary breast and ovarian cancer (HBOC) syndrome (9). BRCA1/2 contribute to homologous recombination (HR) repair, which is the most important mechanism for repairing double-strand breaks in the DNA. Loss of BRCA1/2 function causes homologous recombination repair deficiency (HRD) (9). Apoptosis is induced when PARP is inhibited in cancer cells bearing a loss of BRCA1/2 protein function (10). This killing mechanism is called synthetic lethality. Furthermore, HRD makes the cells sensitive to platinum-based anticancer drugs (10). PARP inhibitors are indicated for HBOC syndrome-related cancers with HRD, including breast, ovarian and prostate cancers (10). It is also indicated for maintenance therapy in platinum-sensitive recurrent ovarian cancer regardless of *BRCA1/2* status (10). Germline *BRCA1/2* mutations were found in 6.7–9.7% of pancreatic cancers, indicating that pancreatic cancer is a phenotype of HBOC syndrome (9). A PARP inhibitor, olaparib (Ola), is also indicated for treating patients with advanced pancreatic cancer with loss-of-function mutations in the germline *BRCA1/2* gene and remaining sensitivity to the platinum-based anticancer drugs (10). We treated a patient with advanced pancreatic cancer that bears a BRCA2 p.I3169M fs^*^48 germline variant. BRCA2 p.I3169M fs^*^48 has a frame-shift substitution from isoleucine residue to methionine residue at codon 3169, adding unrelated 48 amino acids (AA) to the C-terminus of BRCA2 p.I3169M. Hence, the BRCA2 protein is truncated retaining ~93% of the AA of wild-type BRCA2. However, several functional domains, including the nuclear localization signal (NLS), the Cyclin-dependent kinase 2 (CDK2) phosphorylation site at S3291 and the TR2 domain which binds Rad51 located in the C-terminus, are lacking (9). The largest database named BRCA exchange reported the BRCA2 p.I3169M fs^*^48 variant as pathogenic but with no clinical information (11). Furthermore, the drug sensitivity of this variant is unavailable in the literature.

It is necessary to develop a functional assay to precisely evaluate cancer-related genetic variants. MyChoice® (Myriad Genetics, Inc., Salt Lake City, UT, USA) allows to assess simultaneously genomic instability and identify the *BRCA1/2* gene variants. However, its use for pancreatic cancer analysis has not been approved. Other methods are being developed, but they are not suitable for the clinic yet. Furthermore, interpretation based on the primary genomic structure of a variant is often insufficient to predict the variant function. For example, it has been reported that the risk of developing cancer varies among individuals with a *BRCA2* variant. In addition, mutation cluster regions are different between breast and ovarian cancers: mutations in the N-terminus and the C-terminus regions of BRCA2 increase the risk of developing breast cancers, whereas mutations in the central region have been linked with ovarian cancers (12). The relationship between genotype and carcinogenesis may vary for each variant. Furthermore, phenotypes such as carcinogenicity (also called penetrance) and response to some drugs might differ among variants. Thus, genome informatics is very important, and data linking each variant to clinical information are currently being collected such as in the Center for Cancer Genomics and Advanced Therapeutics database in Japan (13). Therefore, the therapeutic response of the pathogenic BRCA2 p.I3169M fs^*^48 variant to platinum and a PARP inhibitor is worth reporting.

## Case presentation

**Figure 1:**
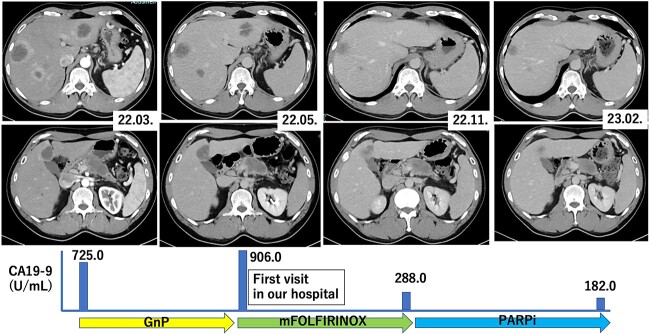
The clinical course of the case. GnP: gemcitabine plus nab-paclitaxel; mFOLFIRINOX: modified FOLFIRINOX composed of oxaliplatin, irinotecan and continuous 5-fluorouracil infusion; PARPi: poly ADP ribose polymerase inhibitor.

A 46-year-old male patient complained the lower abdominal pain and abdominal distension in December 2021, and he visited a local doctor in March 2022. Abdominal ultrasonography and computed tomography (CT) revealed multiple tumors in the pancreas and liver, as well as multiple swollen lymph nodes. He was clinically diagnosed with unresectable pancreatic cancer with multiple liver and lymph node metastases, and systemic chemotherapy with GnP was initiated in March 2022 (Fig. 1). This patient had one measurable primary lesion, two measurable target liver metastases and many unmeasurable lymph node metastases. Retrospective CT scan evaluation with the revised response evaluation criteria in solid tumors version 1.1 (RECIST) revealed a tumoral enlargement to 100.2% of the size before treatment with GnP. In addition, considering the other lesions, the cancer was categorized as stable disease (SD) (14). The patient’s former physicians estimated that GnP was ineffective (Fig. 1), consequently, the patient consulted our department.

In May 2022, the laboratory data were as follows: white blood cells: 6000/μL, neutrophiles: 3700/μL, hemoglobin: 13.0 g/dL, platelets: 21.8 × 10^4^/μL, albumin: 4.7 g/dL, aspartate aminotransferase: 27 U/L, alanine aminotransferase: 39 U/L, total-bilirubin (T-Bil): 1.6 mg/dL, serum creatinine: 0.9 mg/dL, serum Na: 141 mEq/L, serum K: 4.6 mEq/L, serum corrected Ca: 10.0 mg/dL, serum Cl: 102 mEq/L, C-reactive protein: 0.40 mg/dL, carcinoembryonic antigen: 1.8 ng/mL, carbohydrate antigen 19-9 (CA19-9): 906 U/mL. The laboratory data were almost normal except for the T-Bil. The *UGT1A1* gene was ^*^6/^*^6 homozygous, indicating constitutional hyperbilirubinemia.

**Figure 2:**
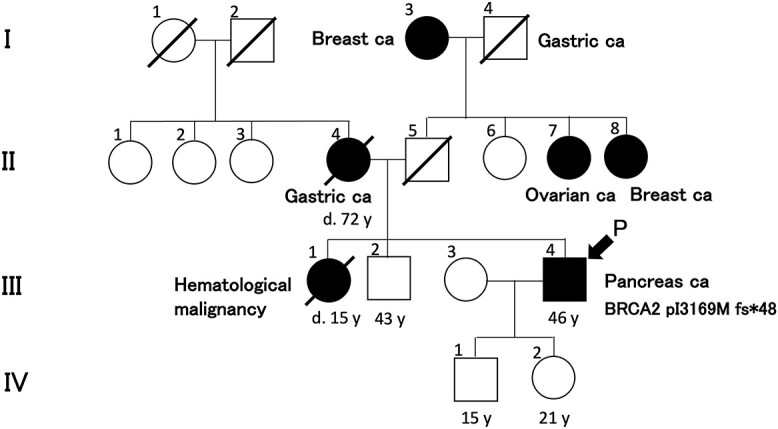
Family tree of this case. Breast Ca: breast cancer, gastric Ca: gastric cancer, ovarian Ca: ovarian cancer, pancreatic Ca: pancreatic cancer, P: proband.

**Figure 3:**
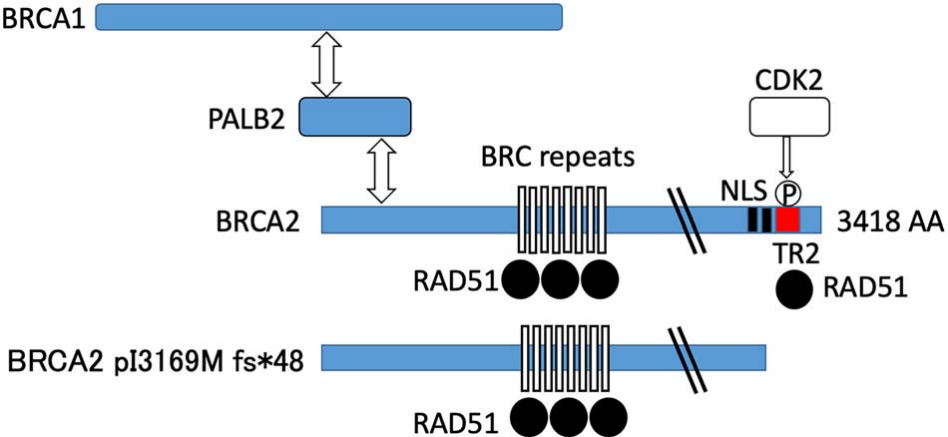
Domain constructions of BRCA2 and BRCA2 pI3169M fs^*^48. BRCA1, PALB2 and BRCA2 form a trimer. BRCA2 can associate RAD51 (indicated by closed circles) via BRC repeats (indicated by open rectangles). BRCA2 pI3169M fs^*^48 lacks nuclea localization signals (NLSs), TR2 domain associating RAD51, and phosphorylation (P) site by CDK2.

His family history revealed two females of the four paternal sibs with breast and ovarian cancers, respectively (Fig. 2, II-7, 8). The paternal grandmother also had breast cancer (Fig. 2, I-3). Furthermore, his younger sister died at the age of 15 years from a hematologic malignancy (Fig. 2, III-1). HBOC was suspected based on these family histories, and germline BRCA analysis (BRACAnalysis®, Myriad Genetics, Inc., Salt Lake City, UT, USA) was performed in April 2022. A germline variant of BRCA2 p.I3169M fs^*^48 was discovered. Then, chemotherapy with modified FOLFIRINOX (mFOLFIRINOX) was started in May 2022 (Fig. 1). He had grade 4 neutropenia and was treated with reduced doses of L-OHP (130 mg, 78% of the standard), IRI (230 mg, 78%) and continuous 5FU infusion (3750 mg, 80%). Two successive CT examinations conducted every 3 months revealed shrinking effects in the primary lesion, metastatic lesions and lymph nodes until November 2022 (Fig. 1). The CT scan evaluation with RECIST indicated that the tumors had shrunk to 76.1% of the pretreatment size. The cancer was determined an SD, considering other lesions.

The CA19-9 level decreased to 288.0 U/mL, which is 20% of the previous level with mFOLFIRINOX treatment (Fig. 1). Tumor shrinkage was maintained for 6 months with mFOLFIRINOX. Then, the treatment was changed to maintenance therapy with oral Ola in December 2022. The adverse event of Ola was slight anorexia alone. A biopsy of a liver metastatic lesion in January 2023 confirmed moderately to poorly differentiated adenocarcinoma of invasive ductal pancreatic carcinoma. However, the number of cells was small and was considered unsuitable for cancer genome testing. CA19-9 further decreased to 182.0 U/mL after switching to Ola (Fig. 1). CT scans every 3 months revealed further shrinkage of the primary tumor, metastases and lymph nodes until July 2023. According to RECIST, the total tumoral size was reduced to 58.2% of the size before treatment with mFOLFIRINOX, and this shrinkage corresponded to a partial response.

## Discussion

Ola is currently used for unresectable advanced pancreatic cancer with HR-D after platinum-based treatment such as FOLFIRINOX (6). A complex consisting of BRCA1, BRCA2, and their connecting protein PALB2 is formed as a trimer and recruited to a double-strand break of DNA (9). BRCA2 in this trimer binds a final effector, thereby exerting the enzymatic activity of homologous recombinase. Furthermore, genes, including *ATM, BARD1, BRIP1, CHEK2, MRE11, RAD50, NBS1, PALB2, RAD51C, RAD51D*, etc., are involved in the HR repair pathway exhibiting a new concept called “BRCAness” (9,15). BRCA2 p.I3169M fs^*^48 discovered in this case lacks functional domains, such as the NLS, the phosphorylation site at S3291 by CDK2 and the TR2 domain. The NLS localizes BRCA2 to the nucleus (16) (Fig. 3).

CDK2-phosphorylated BRCA2 is involved in binding BRCA2 to RAD51, which acts as homologous recombinase. The TR2 domain of BRCA2 is one of the RAD51 binding sites (9). Mainly, we guess that the lacking NLS of BRCA2 cannot go to the DNA double-strand break site in the nucleus, resulting in HR-D. BRCA2 p.I3169M fs^*^48 is listed as a pathogenic variant in the databases due to these structural and presumed functional defects (17). However, the literature described no sensitivity to platinum or PARP inhibitors in pancreatic cancer or other HBOC-related cancers bearing this variant. This case demonstrates that mFOLFIRINOX decreases the size of pancreatic tumors and their metastatic lesions. We evaluated the tumor responses every 3 months using CT, and the second evaluation after mFOLFIRINOX administration confirmed a better outcome than SD. In the pivotal phase 3 clinical trial of a PARP inhibitor in pancreatic cancer with germline mutations in *BRCA1/2* (POLO study), the drug was to be switched from mFOLFIRINOX to Ola in cases where the tumors remained non-progressive according to RECIST after at least 4 months of mFOLFIRINOX treatment (18). The switch from mFOLFIRINOX to Ola occurred after 6 months in this case.

Tumor shrinkage was further maintained with PARP inhibitors. The mPFS was 7.4 months and the mOS was 18.9 months in the POLO study (18). The PFS in our case is >15 months, which is twice longer than the mPFS in the POLO study (18). These observations indicate that the BRCA2 p.I3169M fs^*^48 is a predictive sensitivity marker of platinum-based drugs and PARP inhibitors. The BRCA2 p.I3169M fs^*^48 variant retains 93% of the normal BRCA2, but the C-terminus domain, especially the NLS, might be essential to keep the normal BRCA2 function. An *in vitro* experiment revealed that the deletion of the C-terminus 224 AA of 3418 AA BRCA2 remaining N-terminus 3194 AA could not localize in the nucleus (13,19).

Rebbeck et al. reported an association of the type and location of *BRCA2* mutations with the risk of developing breast and ovarian cancers (12). In *BRCA2*, multiple breast cancer cluster regions (BCCRs) spanning c.1 to c.596, c.772 to c.1806 and c.7394 to c.8904 were identified. In addition, three ovarian cancer cluster regions (OCCRs) spanning c.3249 to c.5681 were found. However, the BCCRs and OCCRs never overlapped, and they concluded that breast and ovarian cancer risks varied according to the type and location of *BRCA2* mutations. Furthermore, Rafnar et al. reported that the BRCA2 K3326^*^ allele does not affect the level of the transcripts and is expressed to the same extent as the wild-type allele (20). Ten out of 17 individuals homozygous for this variant allele did not develop any cancer, whereas three had skin cancers, two had lung cancers, one had colorectal cancer and one presented pancreatic carcinoid. None developed breast or ovarian cancer. BRCA2 K3326 is localized on the C-terminus side of BRCA2 I3169. They concluded that the BRCA2 K3326^*^ allele might encode a variant protein retaining the DNA repair capabilities in tissues responding to hormones such as breast and ovary. These data indicate that some pathogenic variants might not inevitably induce cancer. Like BRCA2 K3326^*^, the BRCA2 p.I3169M fs^*^48 variant retains almost all functional domains of BRCA2. If BRCA2 p.I3169M fs^*^48 variant had some DNA repair capabilities, cancers with this variant might be insensitive to platinum or PARP inhibitors.

However, it was revealed that pancreas cancer with BRCA2 p.I3169M fs^*^48 variant is practically sensitive to both platinum and PARP inhibitors in our case.

The PARP inhibitor resistance might be also ascertained in the future in this case. In particular, the re-establishment of replication fork stability and HR reactivation are known for PARP inhibitor resistance (9,21). Resistance has not been established yet in our case, and the possibility of resistance remains unknown. We must continue to monitor the situation. The tumor sample obtained by fine needle biopsy of liver metastases was small and inadequate for the cancer genome test. Re-biopsy should be considered if the liver metastases might regrow and become much larger. Furthermore, the efficacy of immune checkpoint inhibitors against BRCA2 alteration has been reported (22). We hope the new approval of some effective drugs for this case during the disease is under control. Furthermore, the existence of the BRCA2 pI3169M fs^*^48 in the germline of his younger brother (Fig. 2, III-2) and his two children (Fig. 2, IV-1, IV-2) should be examined under the necessary genetic counseling following their consent.

## Conclusion

Examining family histories in patients with pancreatic cancer is very important. Platinum might be effective if they have *BRCA1/2* germline variants. Pancreatic cancer that bears germline BRCA2 p.I3169M fs^*^48 variant is sensitive to platinum and PARP inhibitors.
